# Remdesivir and chloroquine effectively inhibit the recently emerged novel coronavirus (2019-nCoV) in vitro

**DOI:** 10.1038/s41422-020-0282-0

**Published:** 2020-02-04

**Authors:** Manli Wang, Ruiyuan Cao, Leike Zhang, Xinglou Yang, Jia Liu, Mingyue Xu, Zhengli Shi, Zhihong Hu, Wu Zhong, Gengfu Xiao

**Affiliations:** 1 State Key Laboratory of Virology, Wuhan Institute of Virology, Center for Biosafety Mega-Science, Chinese Academy of Sciences, 430071 Wuhan, China; 2 National Engineering Research Center for the Emergency Drug, Beijing Institute of Pharmacology and Toxicology, 100850 Beijing, China

**Keywords:** Cell biology, Molecular biology

## Dear Editor,

In December 2019, a novel pneumonia caused by a previously unknown pathogen emerged in Wuhan, a city of 11 million people in central China. The initial cases were linked to exposures in a seafood market in Wuhan.^1^ As of January 27, 2020, the Chinese authorities reported 2835 confirmed cases in mainland China, including 81 deaths. Additionally, 19 confirmed cases were identified in Hong Kong, Macao and Taiwan, and 39 imported cases were identified in Thailand, Japan, South Korea, United States, Vietnam, Singapore, Nepal, France, Australia and Canada. The pathogen was soon identified as a novel coronavirus (2019-nCoV), which is closely related to sever acute respiratory syndrome CoV (SARS-CoV).^2^ Currently, there is no specific treatment against the new virus. Therefore, identifying effective antiviral agents to combat the disease is urgently needed.

An efficient approach to drug discovery is to test whether the existing antiviral drugs are effective in treating related viral infections. The 2019-nCoV belongs to *Betacoronavirus* which also contains SARS-CoV and Middle East respiratory syndrome CoV (MERS-CoV). Several drugs, such as ribavirin, interferon, lopinavir-ritonavir, corticosteroids, have been used in patients with SARS or MERS, although the efficacy of some drugs remains controversial.^3^ In this study, we evaluated the antiviral efficiency of five FAD-approved drugs including ribavirin, penciclovir, nitazoxanide, nafamostat, chloroquine and two well-known broad-spectrum antiviral drugs remdesivir (GS-5734) and favipiravir (T-705) against a clinical isolate of 2019-nCoV in vitro.

**Fig. 1:**
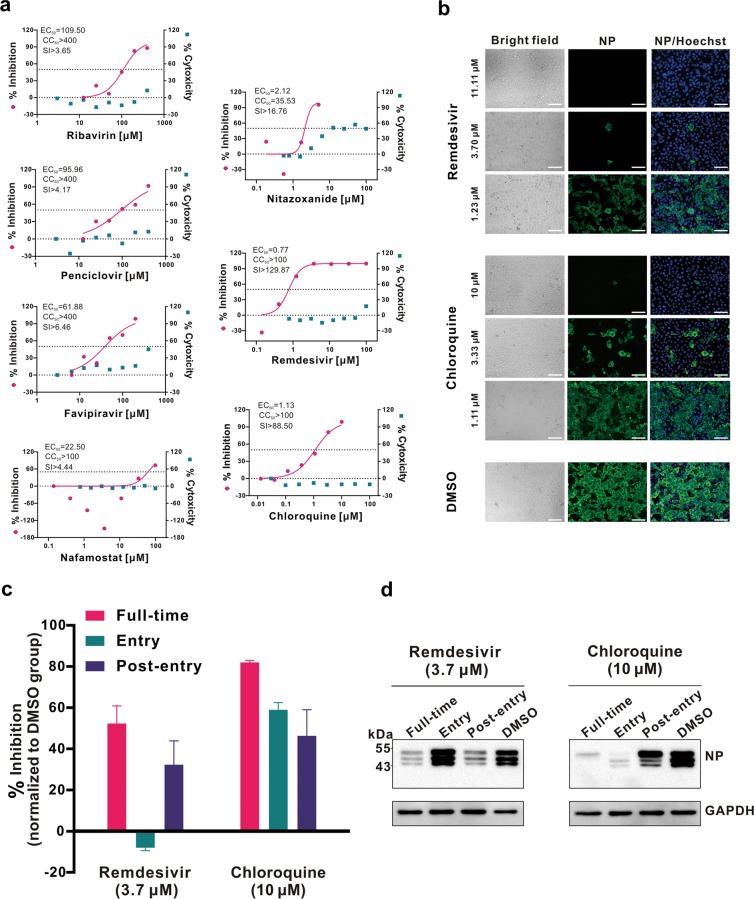
The antiviral activities of the test drugs against 2019-nCoV in vitro. **a** Vero E6 cells were infected with 2019-nCoV at an MOI of 0.05 in the treatment of different doses of the indicated antivirals for 48 h. The viral yield in the cell supernatant was then quantified by qRT-PCR. Cytotoxicity of these drugs to Vero E6 cells was measured by CCK-8 assays. The left and right *Y*-axis of the graphs represent mean % inhibition of virus yield and cytotoxicity of the drugs, respectively. The experiments were done in triplicates. **b** Immunofluorescence microscopy of virus infection upon treatment of remdesivir and chloroquine. Virus infection and drug treatment were performed as mentioned above. At 48 h p.i., the infected cells were fixed, and then probed with rabbit sera against the NP of a bat SARS-related CoV^2^ as the primary antibody and Alexa 488-labeled goat anti-rabbit IgG (1:500; Abcam) as the secondary antibody, respectively. The nuclei were stained with Hoechst dye. Bars, 100 μm. **c** and **d** Time-of-addition experiment of remdesivir and chloroquine. For “Full-time” treatment, Vero E6 cells were pre-treated with the drugs for 1 h, and virus was then added to allow attachment for 2 h. Afterwards, the virus–drug mixture was removed, and the cells were cultured with drug-containing medium until the end of the experiment. For “Entry” treatment, the drugs were added to the cells for 1 h before viral attachment, and at 2 h p.i., the virus–drug mixture was replaced with fresh culture medium and maintained till the end of the experiment. For “Post-entry” experiment, drugs were added at 2 h p.i., and maintained until the end of the experiment. For all the experimental groups, cells were infected with 2019-nCoV at an MOI of 0.05, and virus yield in the infected cell supernatants was quantified by qRT-PCR **c** and NP expression in infected cells was analyzed by Western blot **d** at 14 h p.i.

Standard assays were carried out to measure the effects of these compounds on the cytotoxicity, virus yield and infection rates of 2019-nCoVs. Firstly, the cytotoxicity of the candidate compounds in Vero E6 cells (ATCC-1586) was determined by the CCK8 assay. Then, Vero E6 cells were infected with nCoV-2019BetaCoV/Wuhan/WIV04/2019^2^ at a multiplicity of infection (MOI) of 0.05 in the presence of varying concentrations of the test drugs. DMSO was used in the controls. Efficacies were evaluated by quantification of viral copy numbers in the cell supernatant via quantitative real-time RT-PCR (qRT-PCR) and confirmed with visualization of virus nucleoprotein (NP) expression through immunofluorescence microscopy at 48 h post infection (p.i.) (cytopathic effect was not obvious at this time point of infection). Among the seven tested drugs, high concentrations of three nucleoside analogs including ribavirin (half-maximal effective concentration (EC_50_) = 109.50 μM, half-cytotoxic concentration (CC_50_) > 400 μM, selectivity index (SI) > 3.65), penciclovir (EC_50_ = 95.96 μM, CC_50_ > 400 μM, SI > 4.17) and favipiravir (EC_50_ = 61.88 μM, CC_50_ > 400 μM, SI > 6.46) were required to reduce the viral infection (Fig. 1a and Supplementary information, Fig. S1). However, favipiravir has been shown to be 100% effective in protecting mice against Ebola virus challenge, although its EC_50_ value in Vero E6 cells was as high as 67 μM,^4^ suggesting further in vivo studies are recommended to evaluate this antiviral nucleoside. Nafamostat, a potent inhibitor of MERS-CoV, which prevents membrane fusion, was inhibitive against the 2019-nCoV infection (EC_50_ = 22.50 μM, CC_50_ > 100 μM, SI > 4.44). Nitazoxanide, a commercial antiprotozoal agent with an antiviral potential against a broad range of viruses including human and animal coronaviruses, inhibited the 2019-nCoV at a low-micromolar concentration (EC_50_ = 2.12 μM; CC_50_ > 35.53 μM; SI > 16.76). Further in vivo evaluation of this drug against 2019-nCoV infection is recommended. Notably, two compounds remdesivir (EC_50_ = 0.77 μM; CC_50_ > 100 μM; SI > 129.87) and chloroquine (EC_50_ = 1.13 μM; CC_50_ > 100 μM, SI > 88.50) potently blocked virus infection at low-micromolar concentration and showed high SI (Fig. 1a, b).

Remdesivir has been recently recognized as a promising antiviral drug against a wide array of RNA viruses (including SARS/MERS-CoV^5^) infection in cultured cells, mice and nonhuman primate (NHP) models. It is currently under clinical development for the treatment of Ebola virus infection.^6^ Remdesivir is an adenosine analogue, which incorporates into nascent viral RNA chains and results in pre-mature termination.^7^ Our time-of-addition assay showed remdesivir functioned at a stage post virus entry (Fig. 1c, d), which is in agreement with its putative anti-viral mechanism as a nucleotide analogue. Warren et al. showed that in NHP model, intravenous administration of 10 mg/kg dose of remdesivir resulted in concomitant persistent levels of its active form in the blood (10 μM) and conferred 100% protection against Ebola virus infection.^7^ Our data showed that EC_90_ value of remdesivir against 2019-nCoV in Vero E6 cells was 1.76 μM, suggesting its working concentration is likely to be achieved in NHP. Our preliminary data (Supplementary information, Fig. S2) showed that remdesivir also inhibited virus infection efficiently in a human cell line (human liver cancer Huh-7 cells), which is sensitive to 2019-nCoV.^2^

Chloroquine, a widely-used anti-malarial and autoimmune disease drug, has recently been reported as a potential broad-spectrum antiviral drug.^8,9^ Chloroquine is known to block virus infection by increasing endosomal pH required for virus/cell fusion, as well as interfering with the glycosylation of cellular receptors of SARS-CoV.^10^ Our time-of-addition assay demonstrated that chloroquine functioned at both entry, and at post-entry stages of the 2019-nCoV infection in Vero E6 cells (Fig. 1c, d). Besides its antiviral activity, chloroquine has an immune-modulating activity, which may synergistically enhance its antiviral effect in vivo. Chloroquine is widely distributed in the whole body, including lung, after oral administration. The EC_90_ value of chloroquine against the 2019-nCoV in Vero E6 cells was 6.90 μM, which can be clinically achievable as demonstrated in the plasma of rheumatoid arthritis patients who received 500 mg administration.^11^ Chloroquine is a cheap and a safe drug that has been used for more than 70 years and, therefore, it is potentially clinically applicable against the 2019-nCoV.

Our findings reveal that remdesivir and chloroquine are highly effective in the control of 2019-nCoV infection in vitro. Since these compounds have been used in human patients with a safety track record and shown to be effective against various ailments, we suggest that they should be assessed in human patients suffering from the novel coronavirus disease.

## Supplementary information

Supplementary information, Materials and Figures
