# Supplementary material for: Remdesivir and chloroquine effectively inhibit the recently emerged novel coronavirus (2019-nCoV) in vitro
Source: Cell Res. 2020 Feb 4;30(3):269–71. doi: 10.1038/s41422-020-0282-0 (PMC7054408; doi:10.1038/s41422-020-0282-0)
Supplement: Supplementary file 1 — Supplementary information, Materials and Figures [file 41422_2020_282_MOESM1_ESM.pdf]

Supplementary information, Materials and Methods

Cells, virus and antivirals

African green monkey kidney Vero E6 cell line was obtained from American Type Culture Collection (ATCC, no. 1586) and maintained in minimum Eagle's medium (MEM; Gibco Invitrogen) supplemented with 10% fetal bovine serum (FBS; Gibco Invitrogen) at 37 °C in a humidified atmosphere of 5% CO_2. Human liver cancer Huh7 cell line was cultured in minimum Eagle's medium (Dulbecco's modified Eagle's medium; Gibco Invitrogen) supplemented with 10% FBS at 37 °C with 5% CO_2 atmosphere.

A clinical isolate nCoV-2019BetaCoV/Wuhan/WIV04/2019 [1] was propagated in Vero E6 cells, and viral titer was determined by 50% tissue culture infective dose ($TCID_{50}$) using immunofluorescence assay (the below description). All the infection experiments were performed in a biosafety level-3 (BLS-3) laboratory.

Ribavirin (Cat no. S2504), penciclovir (Cat no. S4184), favipiravir (Cat no. S7975) and nitazoxanide (Cat no. S1627) were purchased from Selleck Chemicals. Chloroquine was obtained from Sigma-Aldrich (Cat no. C6628). Remdesivir (Cat no. HY-104077) and nafamostat (Cat no. HY-B0190) were purchased from MedChemExpress.

Evaluation of antiviral activities of the drugs

The cytotoxicity of the tested drugs on Vero E6 or Huh-7 Cells were determined by CCK8 assays (Beyotime, China). To evaluate the antiviral efficacy of these drugs, Vero E6 or Huh-7 cells were cultured overnight in 48-well cell-culture petridish with a density of 5×10^4 cells/well. Cells were pre-treated with the different doses of the indicated antivirals for 1 h, and the virus (MOI of 0.05) was subsequently added to allow infection for 2 h. Then, the virus-drug mixture was removed and cells were further cultured with fresh drug-containing medium. At 48 h p.i., the cell supernatant was collected and lysed in lysis buffer (Takara, Cat no. 9766) for further quantification analysis.

Viral RNA extraction and quantitative real-time RT-PCR (qRT-PCR)

One hundred microliter cell culture supernatant was harvested for viral RNA extraction using the MiniBEST Viral RNA/DNA Extraction Kit (Takara, Cat no. 9766) according to the manufacturer's instructions. RNA was eluted in 30 μL RNase-free water. Reverse transcription was performed with a PrimeScript RT Reagent Kit with gDNA Eraser (Takara, Cat no. RR047A) and qRT-PCR was performed on StepOne Plus Real-time PCR system (Applied Biosystem) with TB Green Premix Ex Taq II (Takara Cat no.RR820A). Briefly, 3 μL total RNA was first digested with gDNA eraser to remove contaminated DNA and then the first-strand cDNA was synthesized in 20 μL reaction and 2 μL cDNA was used as template for quantitative PCR. Receptor binding domain (RBD) of spike gene was amplified by PCR from the cDNA template with primers: RBD-F:

5’-GCTCCATGGCCTAATATTACAAACTTGTGCC3’; RBD-R: 5’-TGCTCTAGACTCAAGTGTCTGTGGATCAC-3’, and cloned into pMT/BiP/V5-His vector (Invitrogen) and used as the plasmid standard after its identity was confirmed by sequencing. A standard curve was generated by determination of copy numbers from serially dilutions (10^3-10^9 copies) of the plasmid. The primers used for quantitative PCR were RBD-qF1: 5’-CAATGGTTTAACAGGCACAGG-3’ and RBD-qR1:5’-CTCAAGTGTCTGTGGATCACG-3. [1] PCR amplification was performed as follows: 95 °C for 5 min followed by 40 cycles consisting of 95 °C for 15 s, 54 °C for 15 s, 72 °C for 30 s. The dose-response curves were plotted form viral RNA copies *versus* the drug concentrations by using GraphPad Prism 6 software.

Immunofluorescence microscopy

To detect viral protein expression in Vero E6 cells or Huh-7 cells, cells were fixed with 4% paraformaldehyde and permeabilized with 0.5% Triton X-100. Then the cells were blocked with 5% bovine serum albumin (BSA) at room temperature for 2 h. The cells were further incubated with the primary antibody (a polyclonal antibody against viral nucleocapsid protein [abbreviated as anti-NP] of a bat SARS-related CoV, 1:1000 dilution) [1] for 2 h, followed by incubation with the secondary antibody (Alexa 488-labeled goat anti-rabbit [1:500; Abcam]). The nuclei were stained with Hoechst 33258 dye (Beyotime, China). The images were taken by fluorescence microscopy.

Time-of-addition experiment of remdesivir and chloroquine

The remdesivir (3.7 μM) and chloroquine (10 μM) were used for the time-of-addition experiment. Vero E6 cells (5×10^4 cells/well) were treated with remdesivir, chloroquine or DMSO at different stages of virus infection. For "Full-time" treatment, Vero E6 cells were pre-treated with the drugs for 1 h prior to virus infection, followed by incubation with virus for 2 h in the presence of the drugs. Then, the virus-drug mixture was removed. Cells were washed with PBS, and further cultured with drug-containing medium until the end of the experiment. For "Entry" treatment, the drugs were added to the cells for 1 h before virus infection, and maintained during the 2-h viral attachment process. Then, the virus-drug mixture was replaced with fresh culture medium without drugs till the end of the experiment. For "Post-entry" experiment, virus was added to the cells to allow infection for 2 h, and then virus-containing supernatant was replaced with drug-containing medium until the end of the experiment. The experimental condition of the DMSO-treatment group was consistent with that of the "Full-time" group. For all the experimental groups, cells were infected with virus at an MOI of 0.05, and at 14 h p.i., cell supernatant and cell lysates were collected for qRT-PCR and Western blot analysis, respectively.

Western blot analysis

For Western blot analysis, protein samples were separated on 12% SDS-PAGE and then transferred onto polyvinylidene difluoride (PVDF) membranes (Millipore). After being blocked with 5% BSA in TBS buffer containing 0.05% Tween 20, the blot was probed with the anti-NP antibody (1:2000 dilution) and the horseradish peroxidase

(HRP)-conjugated Goat Anti-Rabbit IgG (Proteintech, China) as the first and the secondary antibody, respectively. Protein bands were detected by SuperSignal West Pico Chemiluminescent substrate (Pierce).

References:

1. Zhou, P. et al. *BioRxiv preprint* https://doi.org/10.1101/2020.01.22.914952 (2020).

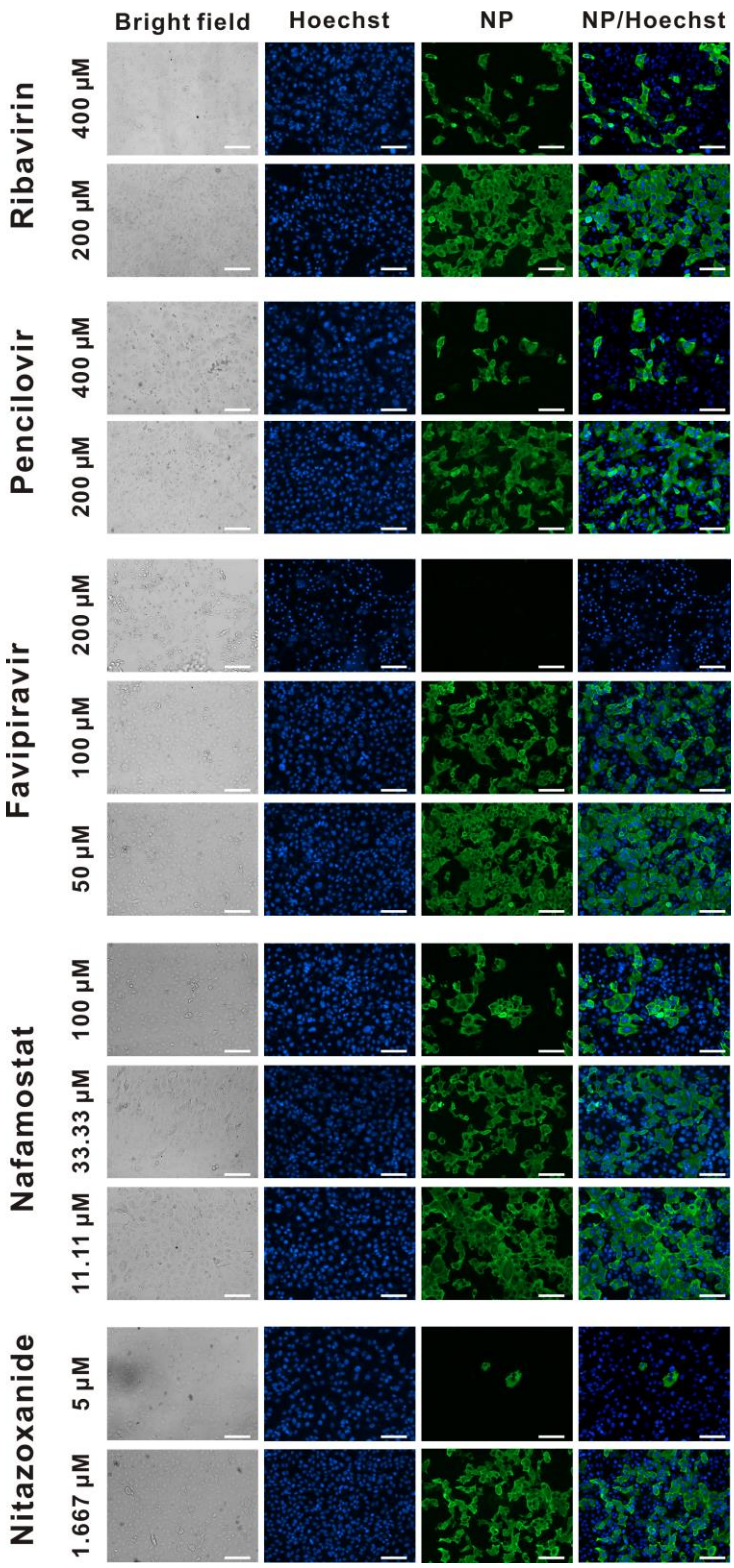

Supplementary information, Fig. S1 Immunofluorescence microscopy of virus infection upon treatment of ribavirin, penciclovir, favipiravir, nafamostat, and nitazoxanide. Vero E6 cells were infected with 2019-nCoV at an MOI of 0.05 in the treatment of different concentrations of the indicated antivirals for 48 h. Then the infected cells were subjected to immunofluorescence analysis by using anti-NP rabbit sera. The nuclei were stained with Hoechst dye. Bars, 100 μm.

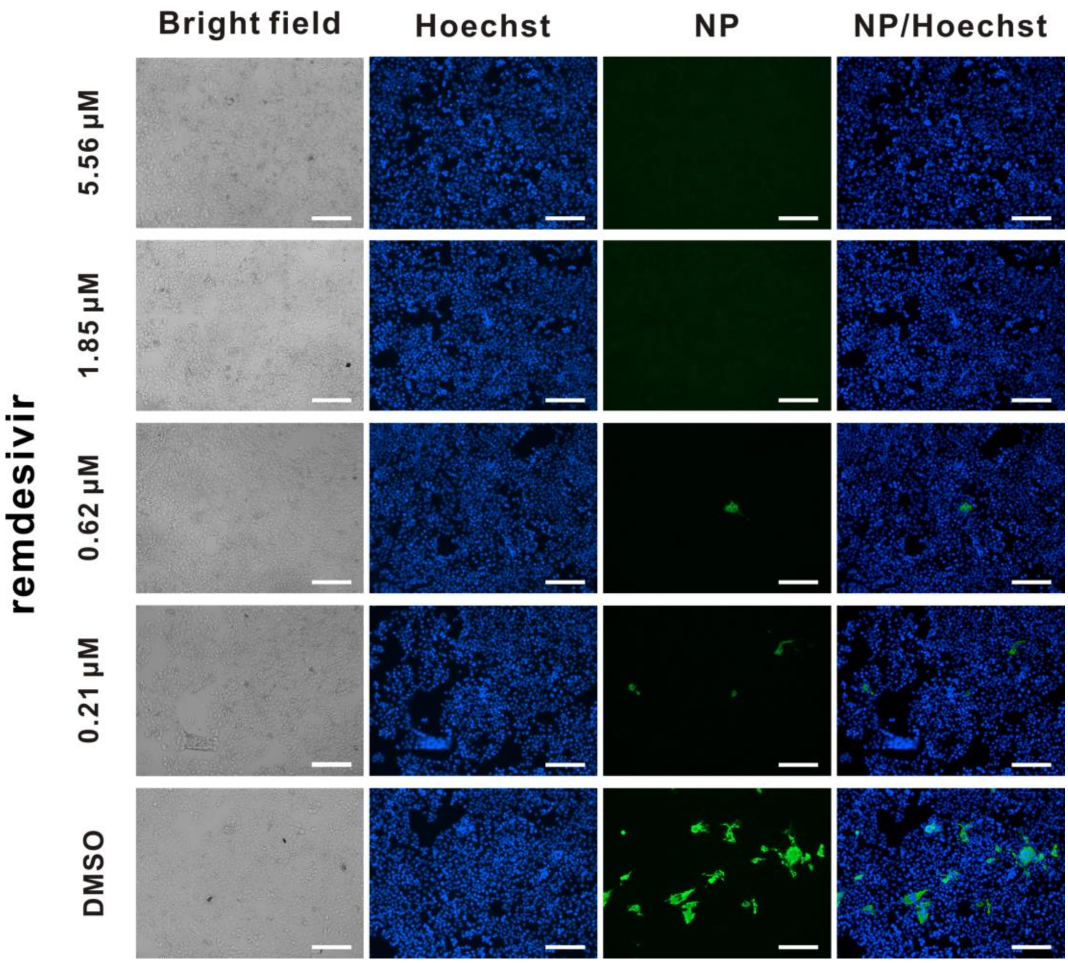

Supplementary information, Fig. S2 The antiviral activity of remdesivir against 2019-nCoV in human liver cancer Huh7 cells. Huh7 cells were infected with 2019-nCoV at an MOI of 0.1 in the treatment of the indicated concentrations of remdesivir or DMSO. At 48 h p.i., the infected cells were subjected to immunofluorescence microscopy by using anti-NP rabbit sera. The cell nuclei were stained with Hoechst dye. Bars, 200 μm.
